# Enhanced synchronization between prelimbic and infralimbic cortices during fear extinction learning

**DOI:** 10.1186/s13041-021-00884-6

**Published:** 2021-12-11

**Authors:** Mayumi Watanabe, Akira Uematsu, Joshua P. Johansen

**Affiliations:** 1 RIKEN Center for Brain Science, Wako-shi, Saitama Japan; 2 Department of Life Sciences, Graduate School of Arts and Sciences, The University of Tokyo, Tokyo, Japan; 3 Department of Biological Sciences, Graduate School of Science, The University of Tokyo, Tokyo, Japan; 4 International Research Center for Neurointelligence, The University of Tokyo, Tokyo, Japan; 5 RIKEN Center for Brain Science, Laboratory for Neural Circuitry of Learning and Memory, 2-1 Hirosawa, Wako-shi, Saitama 351-0198 Japan

**Keywords:** Fear extinction, Medial prefrontal cortex, Electrophysiology, Oscillation

## Abstract

The ability to extinguish aversive memories when they are no longer associated with danger is critical for balancing survival with competing adaptive demands. Previous studies demonstrated that the infralimbic cortex (IL) is essential for extinction of learned fear, while neural activity in the prelimbic cortex (PL) facilitates fear responding and is negatively correlated with the strength of extinction memories. Though these adjacent regions in the prefrontal cortex maintain mutual synaptic connectivity, it has been unclear whether PL and IL interact functionally with each other during fear extinction learning. Here we addressed this question by recording local field potentials (LFPs) simultaneously from PL and IL of awake behaving rats during extinction of auditory fear memories. We found that LFP power in the fast gamma frequency (100–200 Hz) in both PL and IL regions increased during extinction learning. In addition, coherency analysis showed that synchronization between PL and IL in the fast gamma frequency was enhanced over the course of extinction. These findings support the hypothesis that interregional interactions between PL and IL increase as animals extinguish aversive memories.

**Supplementary Information:**

The online version contains supplementary material available at 10.1186/s13041-021-00884-6.

## Main text

Extinguishing aversive emotional memories recruits cortical and subcortical neural circuits which actively inhibit the expression of emotional responses in a context dependent way. For example, during auditory fear conditioning, animals form an association between a tone and an aversive outcome and express defensive behaviors when the tone is presented after learning. However, if the tone is presented repeatedly in the absence of the aversive outcome, animals extinguish their defensive responses. This learning process is termed fear extinction. Although extinction memories can be maintained for long periods, under certain conditions they can spontaneously recover with the passage of time, reemerge when animals are placed in a different context from the one in which they were extinguished or are re-exposed to the aversive event. Accumulated evidence has revealed that the infralimbic cortex (IL), a subregion of medial prefrontal cortex (mPFC), plays an essential role in extinction learning [1, 2]. For example, neural activity in IL is required for formation of long-term extinction memories through its projections to the amygdala [3–5]. By contrast, the prelimbic cortex (PL), another mPFC subregion, is important for expression of learned fear [6], and neural activity in the PL is negatively correlated with the strength of extinction memory [7, 8].

These findings suggest that PL and IL mediate opposing control of defensive responses independently. Contrasting with this idea, some evidence suggests possible interactions between PL and IL during fear extinction learning. Anatomical and electrophysiological studies showed that PL and IL maintain mutual synaptic connections [9–11]. Furthermore, an electrophysiology study in anesthetized rats demonstrated increased cross-correlations of PL and IL unit activity during CS exposure [12], though this was not linked to behavioral extinction possibly because of the anesthetized preparation and the use of low numbers of CS presentations. Moreover, a recent study demonstrated that excitatory inputs from PL to IL facilitate extinction of conditioned fear memory [11]. Despite this suggestive evidence, it remains unclear whether functional interactions between PL and IL occur during fear extinction learning.

**Fig. 1:**
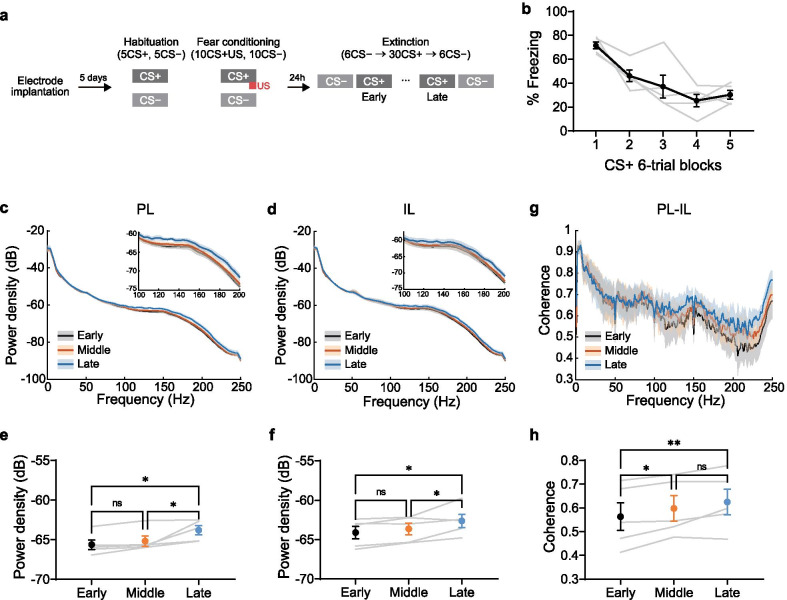
Enhancement of LFP synchronization between PL and IL during fear extinction learning. **a** Experimental protocol. **b** Freezing response to CS+ during extinction learning session. Black line indicates average of all rats and gray lines indicate individual data (n = 5). **c**, **d** LFP power spectra in PL (**c**) and IL (**d**) during CS+ presentation in early (black), middle (orange) and late (blue) phases of extinction learning. Solid lines and shaded areas indicate average and standard error of the mean (SEM) across animals, respectively. Insets show zoomed-in view of the power spectra. **e**, **f** Power density averaged over fast gamma frequency (100–200 Hz) in PL (**e**) and IL (**f**). **g** Coherence between PL and IL during CS+ presentation in early (black), middle (orange) and late (blue) phases of extinction learning. **h** Coherence between PL and IL averaged over fast gamma frequency (100–200 Hz). * p < 0.05; ** p < 0.01; One-way repeated measures ANOVA and post-hoc Newman–Keuls test. Each error bar indicates SEM across animals. The results of the statistical analyses are described in Additional file 1

To examine possible interactions between the PL and IL during fear extinction learning, we implanted electrodes in rats and trained them in differential fear conditioning and extinction (Fig. 1a, Additional file 1: Fig. S2). Animals were first habituated to two neutral auditory cues (5 kHz tone pips and 14 kHz continuous tones, each 10-s duration), then conditioned in the same context. During the conditioning session, one tone was paired with footshock (positive conditioned stimulus, CS+), while the other tone was not associated with shock (negative conditioned stimulus, CS−). Rats were presented with the tones without footshock in a novel context to extinguish the fear memory 24 h after fear conditioning. All rats showed freezing response to CS+ in the beginning of the extinction session, followed by a reduction of freezing during extinction learning (Fig. 1b).

During the extinction session, we recorded local field potentials (LFPs) simultaneously from PL and IL through chronically implanted tungsten wires (Additional file 1: Fig. S1). First we tested whether extinction learning influenced the power of oscillations at different frequencies. Comparison of LFP power spectra during the CS+ presentation showed that fast gamma oscillations (100–200 Hz) were enhanced during extinction learning in both PL (Fig. 1c) and IL (Fig. 1d). Accordingly, LFP power in fast gamma frequency (averaged over 100–200 Hz) was significantly higher in the late phase of extinction compared with the early and middle phases (Fig. 1e, f). During the baseline period before CS+ presentation, fast gamma power in the PL was also increased in the late phase of extinction, while fast gamma power in the IL was not (Additional file 1: Fig. S3a, b). Fast gamma power during CS− presentation did not differ in PL or IL when comparing early to late extinction (Additional file 1: Fig. S3c, d). These observations demonstrate that gamma oscillations increase in PL and IL during extinction and suggest that the increase of fast gamma power is related to the acquisition of extinction memory and not simply a consequence of the passage of time.

Interregional synchronization of gamma oscillations is considered to reflect increases in functional interactions between brain regions, and it is important for a variety of learning including safety learning [13]. To test whether extinction learning influences interregional synchronization between mPFC subregions, we calculated LFP coherence between PL and IL during CS+ presentation. Comparison between extinction phases showed an increase of coherence in fast gamma frequency over the course of extinction learning (Fig. 1g). Fast gamma coherence during CS+ presentation was significantly enhanced in the middle and late phases compared with the early phase (Fig. 1h). By contrast, coherence in the fast gamma frequency during the pre-CS baseline period or the CS− presentation was not affected by extinction learning (Additional file 1: Fig. S4). This suggests that the extinction induced increase in PL–IL coherence is due to extinction learning and not simply the passage of time.

To test whether fast gamma oscillations were modulated by freezing behavior, we compared fast gamma power and coherence between epochs with low and high levels of freezing. Regardless of the presence of the CS, there was no freezing-related difference in fast gamma power or coherence (Additional file 1: Fig. S5). Here the analysis was focused on the middle phase of extinction session because pre-CS freezing was rarely observed in later trials, but similar results were obtained during CS presentation in the late phase (data not shown).

Although LFP coherence reflects synchronized neuronal activity between PL and IL, it cannot exclude the possibility of spurious synchronization caused by volume conduction due to the proximity of these regions. To address this concern, we calculated another coherence measure, weighted phase lag index (WPLI), which is a measure of phase synchronization and free from volume conduction or other potential common noise sources [14]. Consistent with the coherence result, WPLI in the fast gamma frequency was significantly increased during extinction (Additional file 1: Fig. S6a). Another potential concern is that the increase of PL–IL interregional synchronization simply reflects the enhancement of local fast gamma oscillations. Although the fact that the PL–IL synchronization increased earlier in extinction than the increase in local gamma power (Fig. 1e, f, h) suggested that this was not the case, we examined this further by analyzing synchronization during periods of low and high gamma. We sorted pre-CS baseline periods by local fast gamma power and categorized epochs with weak or strong fast gamma, and found no difference in WPLI between weak and strong fast gamma epochs (Additional file 1: Fig. S6b).

Here we demonstrated that fast gamma power within PL and IL and synchronization between these regions were enhanced during fear extinction learning. These findings demonstrate that interregional communication between PL and IL is strengthened over the course of fear extinction.

Neural synchronization in the fast gamma frequency is thought to enhance the efficacy of depolarization in downstream neurons [15] and contribute to different types of learning [16, 17]. A previous study showed that fast gamma power was increased in mPFC during fear extinction learning, although PL and IL were not distinguished in this analysis [13]. Our results demonstrate that the enhancement of fast gamma oscillation occurs in both PL and IL individually. This may result in strengthened top-down control of downstream regions such as basolateral amygdala. Fast gamma oscillations may also enable regulation of neuromodulatory systems, which participate in fear extinction learning. For example, mPFC projects to the noradrenergic locus coeruleus [18] and projections from locus coeruleus to the IL participate in extinction learning [19, 20]. Furthermore, a recent study demonstrated that fast gamma oscillations in mPFC modulate the firing activity of locus coeruleus noradrenergic neurons [21].

Though our data suggest that synchronization between PL and IL is strengthened during fear extinction learning, the nature of this interaction remains unclear. One underlying mechanism may be through the engagement of excitatory synaptic drive from PL to IL, which is known to facilitate fear extinction learning [11]. Since PL pyramidal neurons are activated during fear retrieval, they may activate IL pyramidal neurons to influence extinction learning. Another potential mechanism for the interaction could be through inhibitory connections from IL to PL. An in vivo optogenetic study showed that stimulation of IL pyramidal neurons suppressed the firing activity of PL pyramidal neurons [10]. This IL-to-PL inhibition may be enhanced through fear extinction and the amount of CS-evoked activity in PL neurons, which is negatively correlated with the strength of extinction memory [7, 8], may reflect this. Further work is required to determine which circuit and coding mechanism underlies the interregional synchronization within mPFC and contributes to the extinction of aversive memories.

## Supplementary Information

**Additional file 1**: Description of data: Material and Methods, Summary of Statistics, Figs. S1–6.

## Data Availability

Data will be made available upon reasonable request.

## Abbreviations

mPFC: Medial prefrontal cortex
PL: Prelimbic cortex
IL: Infralimbic cortex
LFP: Local field potential
CS: Conditioned stimulus
WPLI: Weighted phase lag index
