# Supplementary material for: Enhanced synchronization between prelimbic and infralimbic cortices during fear extinction learning
Source: Mol Brain. 2021 Dec 11;14:175. doi: 10.1186/s13041-021-00884-6 (PMC8666018; doi:10.1186/s13041-021-00884-6)
Supplement: Supplementary file 1 — Additional file 1: Description of data: Material and Methods, Summary of Statistics, Figs. S1–6. [file 13041_2021_884_MOESM1_ESM.docx]

**Additional file 1**

**Materials and Methods**

**Animals**

All experimental procedures were approved by the Animal Care and Use Committees of the RIKEN Center for Brain Science. Male adult Long-Evans rats were used for all experiments. Animals were singly housed on a 12-h light/dark cycle, and food and water were provided ad libitum. All behavioral experiments were performed during the light cycle.

**Surgery**

Rats were anesthetized with isoflurane (5% for induction, 1.5-2.5% for maintenance) and placed in a stereotaxic frame (Kopf Instruments). Tungsten electrodes (~250 kΩ impedance) were implanted in PL (from bregma AP +3.2mm, ML +1.6mm, DV -3.2mm, 8° angle) and IL (from bregma AP +2.7mm ML +1.6mm, DV -5.2mm, 8° angle). The electrodes were secured to the skull with stainless screws and dental cement. Two stainless screws were placed on cerebellum as reference and ground. Rats were allowed five days for post-surgical recovery before the start of behavioral experiments.

**Fear conditioning and extinction**

Fear conditioning and extinction training were conducted in different contexts. Rats were first habituated to handling and tethering with the recording cable in the extinction context for two consecutive days.

Two auditory stimuli were used for conditioned stimuli (CS); 5-kHz pip tone repeated at 2 Hz (0.2s ON, 0.3s OFF) and 14-kHz continuous tone. Total duration of each CS was 10 s. The assignment of the tones to CS+ and CS- was counterbalanced between animals.

On day 1, rats were placed in a sound-isolated chamber and presented auditory tones for habituation (5CS+ and 5CS-, pseudorandom order). Following the habituation session, differential fear conditioning was performed in the same context (10CS+&US and 10CS-, pseudorandom order). One tone (CS+) was paired with a 1-s footshock US (0.5 mA), while the other tone (CS-) was not paired. On day 2, rats were presented with 30CS+ and 12CS- in a different context. CS- was presented at the first and last 6 trials, and CS+ was presented in the middle 30 trials.

Duration of the freezing response during CS presentation was manually scored as a behavioral measure of the strength of fear memories.

**In vivo electrophysiology**

Electrodes were connected to a headstage (Neuralynx Inc.) containing 36 unity-gain operational amplifiers. Local field potentials (LFPs) were sampled at 4 kHz, filtered from 0.1 Hz to 2 kHz and acquired through a Neuralynx data acquisition system. At the end of the experiment, recording sites were verified histologically with electrolytic lesions using 20 s of 20-mA direct current.

**Histology**

To evaluate the location of recording electrodes, rats were intraperitoneally overdosed with 3.0 mL of 25% chloral hydrate and transcardially perfused with 4% paraformaldehyde in PBS. After overnight post-fixation, brains were sliced into 40-µm coronal sections using a cryostat. Electrode locations were verified using a fluorescence microscope. Only rats which had electrode placements in both PL and IL were included in the data analysis.

**Data Analysis**

All analyses of electrophysiological data were performed using MATLAB (Mathworks). All LFP signals were low-pass filtered at 250 Hz and downsampled to 500 Hz. Power density was estimated using Welch’s method. For analysis of fast gamma oscillations, LFP data were band-pass filtered between 100 and 200 Hz using the eegfilt function from the EEGLAB toolbox (Delorme & Makeig 2004). LFP coherence was calculated using the mscohere function from the MATLAB Signal Processing Toolbox. Weighted phase-lag index (WPLI, Vinck et al. 2011) was calculated using a custom MATLAB script.

For comparison between extinction phases, eight CS+ trials were included in each early, middle and late phase and averaged. The number of trials in extinction phase was determined by the observation that animals exhibited robust freezing in the first eight trials. For comparison between freezing levels, middle phase epochs (during CS or pre-CS) were divided by individual median of freezing, and averaged within low and high freezing epochs.

**Statistical analysis**

Statistical analyses were performed using GraphPad Prism (GraphPad Software, Inc.). Two-tailed paired Student’s t tests or one-way repeated measures (RM) ANOVA were used to detect differences between extinction phases. Significant main effects of ANOVA were followed by Newman-Keuls post-hoc tests. The significance level was set at P < 0.05 for all results.

**References**

- Delorme A, Makeig S. EEGLAB: an open source toolbox for analysis of single-trial EEG dynamics including independent component analysis. J Neurosci Methods. Elsevier; 2004;134:9–21.
- Vinck M, Oostenveld R, Van Wingerden M, Battaglia F, Pennartz CMA. An improved index of phase-synchronization for electrophysiological data in the presence of volume-conduction, noise and sample-size bias. Neuroimage. 2011;55:1548–65.

**Summary of Statistics**

| Figure | Data | Sample size | Test | Comparison | Statistics | P-value |
| --- | --- | --- | --- | --- | --- | --- |
| 1e | PL fast gamma power during CS+ | n=5 | One-way RM ANOVA | Extinction phase | F(2,8)=  8.14 | 0.012 |
|  |  |  | post-hoc  Neuman-Keuls | early vs middle |  | 0.36 |
|  |  |  |  | early vs late |  | 0.011 |
|  |  |  |  | middle vs late |  | 0.020 |
| 1f | IL fast gamma power during CS+ | n=5 | One-way RM ANOVA | Extinction phase | F(2,8)=  7.53 | 0.015 |
|  |  |  | post-hoc  Neuman-Keuls | early vs middle |  | 0.26 |
|  |  |  |  | early vs late |  | 0.013 |
|  |  |  |  | middle vs late |  | 0.032 |
| 1h | Fast gamma coherence during CS+ | n=5 | One-way RM ANOVA | Extinction phase | F(2,8)=  9.23 | 0.008 |
|  |  |  | post-hoc  Neuman-Keuls | early vs middle |  | 0.042 |
|  |  |  |  | early vs late |  | 0.007 |
|  |  |  |  | middle vs late |  | 0.099 |
| S3a | PL fast gamma power during preCS | n=5 | paired-t | early vs late | t(4)=3.28 | 0.031 |
| S3b | IL fast gamma power during preCS | n=5 | paired-t | early vs late | t(4)=1.23 | 0.29 |
| S3c | PL fast gamma power during CS- | n=4 (discriminator) | paired-t | early vs late | t(3)=0.82 | 0.47 |
| S3d | IL fast gamma power during CS- | n=4 (discriminator) | paired-t | early vs late | t(3)=0.04 | 0.97 |
| S4a | Fast gamma coherence during preCS | n=5 | paired-t | early vs late | t(4)=1.87 | 0.14 |
| S4b | Fast gamma coherence during CS- | n=4 (discriminator) | paired-t | early vs late | t(4)=0.55 | 0.61 |
| S5a | PL fast gamma power during middle CS+ | n=5 | paired-t | low vs high freezing | t(4)=0.92 | 0.41 |
| S5b | IL fast gamma power during middle CS+ | n=5 | paired-t | low vs high freezing | t(4)=1.71 | 0.16 |
| S5c | Fast gamma coherence during middle CS+ | n=5 | paired-t | low vs high freezing | t(4)=1.33 | 0.25 |
| S5d | PL fast gamma power during middle preCS | n=5 | paired-t | low vs high freezing | t(4)=0.89 | 0.42 |
| S5e | IL fast gamma power during middle preCS | n=5 | paired-t | low vs high freezing | t(4)=1.17 | 0.31 |
| S5f | Fast gamma coherence during middle preCS | n=5 | paired-t | low vs high freezing | t(4)=0.90 | 0.42 |
| S6a | Fast gamma WPLI during CS+ | n=5 | paired-t | early vs late | t(4)=2.83 | 0.047 |
| S6b | Fast gamma WPLI during preCS | n=5 | paired-t | weak vs strong  fast gamma epochs | t(4)=0.85 | 0.44 |


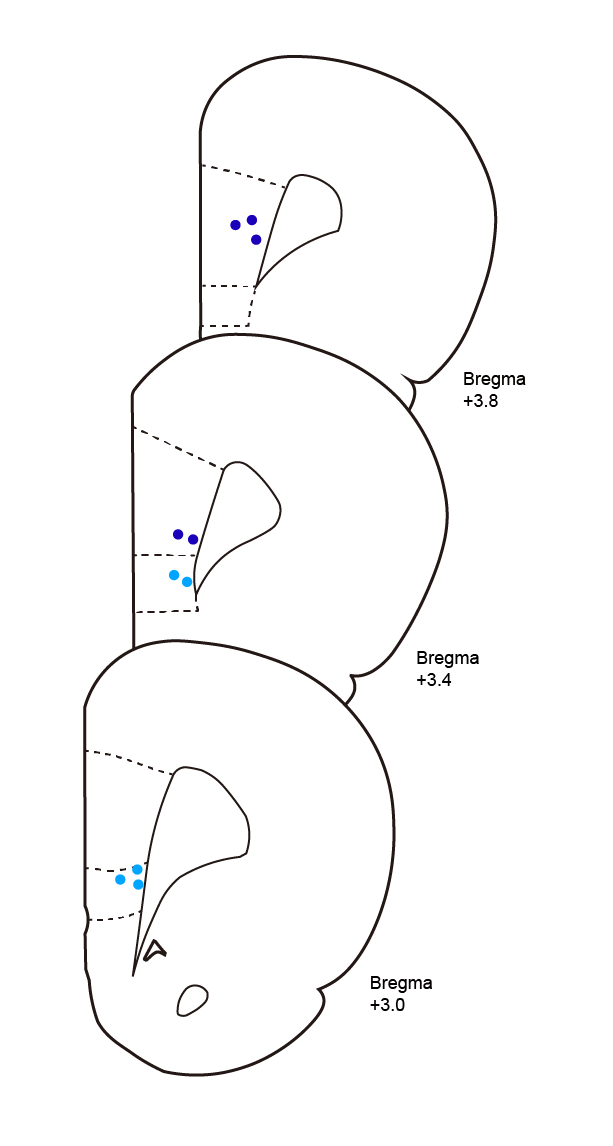


**Figure S1**

**Histological reconstruction of recording sites**

Electrode locations in PL (dark blue) and IL (light blue) were identified by electrolytic lesions as described in Materials and Methods.


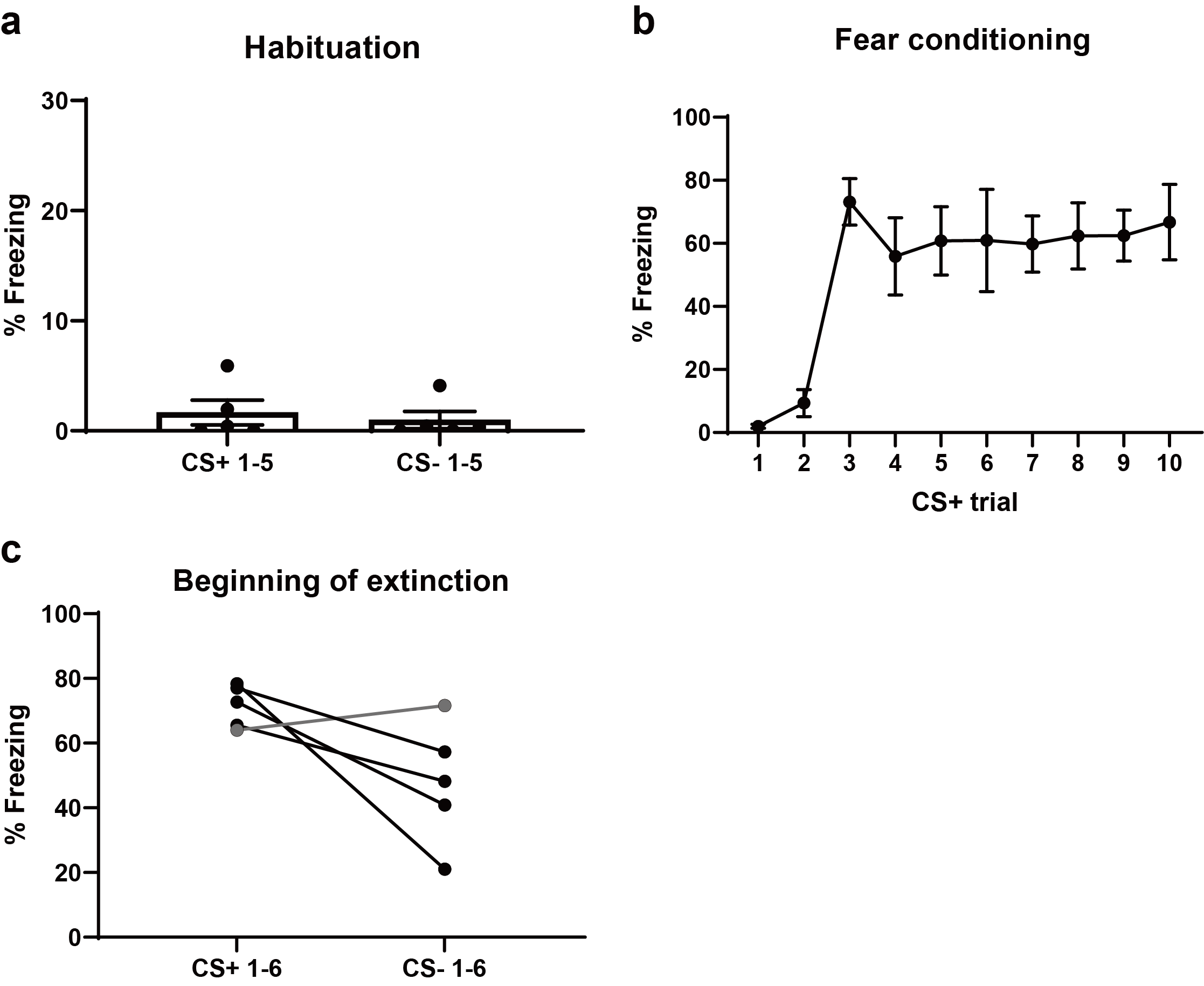


**Figure S2**

**Freezing during habituation, fear conditioning and beginning of extinction**

**a** Freezing responses to CS+ and CS- during habituation session. Errorbars indicate SEM. **b** Freezing responses to CS+ during fear conditioning. **c** Freezing responses to CS+ and CS- in the beginning of extinction session. Four of five rats showed higher freezing to CS+ than CS-. One rat with generalized freezing response (shown in gray) was excluded in the LFP analysis during CS- presentation (Figs. S3 and S4).

**Figure S3**

**Power of fast gamma oscillations during baseline and CS- presentation**

**a–b** Fast gamma power in PL (a) or IL (b) during baseline period before CS+ presentation in early and late extinction. **c–d** Fast gamma power in PL (c) or IL (d) during CS- presentation in early and late extinction (average of each six trials).

**Figure S4**

**Coherence between PL and IL in fast gamma frequency during baseline and CS-**

**a** Fast gamma coherence during baseline period before CS+ presentation in early and late extinction. **b** Fast gamma coherence during CS- presentation in early and late extinction.

**Figure S5**

**Fast gamma power and coherence during high vs. low levels of freezing behavior**

**a-c** Fast gamma power in PL (a) and IL (b) and coherence (c) during CS+ in middle phase of extinction. **d-f** Fast gamma power in PL (d) and IL (e) and coherence (f) during pre-CS baseline period in middle phase of extinction.

**Figure S6**

**Weighted phase lag index (WPLI) between PL and IL in fast gamma frequency**

**a** WPLI during CS+ presentation in early and late extinction. **b** WPLI during weak and strong fast gamma epochs measured in IL (similar results were obtained when measuring gamma in PL).
